# Supplementary material for: Convergent evolution of aerobic fermentation through divergent mechanisms acting on key shared glycolytic genes
Source: EMBO J. 2026 Apr 10;45(10):3540–62. doi: 10.1038/s44318-026-00778-0 (PMC13187154; doi:10.1038/s44318-026-00778-0)
Supplement: Supplementary file 1 — Appendix [file 44318_2026_778_MOESM1_ESM.pdf]

Appendix for “Convergent evolution of aerobic fermentation through divergent mechanisms acting on key shared glycolytic genes”

Table of contents

Appendix Table S1. The primers used in this study. 2

Appendix Table S2. Genetically modified strains made and used for this study. 3

Appendix Table S1. The primers used in this study.

Primer name	Sequence	Purpose
oHLCH064	cctcgaggtcgacggtatcg	pRS426 backbone amplification
oHLCH065	ccggtacccaattcgccctatag	pRS426 backbone amplification
oHLCH482	tagggcgaattgggtaccgg**gcgttagaggtgctacagag**	Amplifying *GAL4* upstream region F with overhang to pRS426
oHLCH483	taaacagatctctagaccta**acaagcctgtgcaggaacca**	Amplifying *GAL4* upstream region R with overhang to *kanMX*
oHLCH484	tggttcctgcacaggcttgt**taggtctagagatctgtttagc**	Amplifying *kanMX* F with overhang to *GAL4* upstream
oHLCH485	gaaggccattgggtcagtaa**ttaagggttctcgagagctcg**	Amplifying *kanMX* R with overhang to *GAL4* downstream
oHLCH486	gagctctcgagaacccttaa**ttactgacccaatggccttc**	Amplifying *GAL4* downstream region F with overhang to *kanMX*
oHLCH487	cgataccgtcgacctcgagg**caccagcaaatatgagccatg**	Amplifying *GAL4* downstream region R with overhang to pRS426
oHLCH004	gattccgactcgtccaacatc	Primer to confirm *kanMX* integration
oHLCH488	ggtgccaacaagaaagactc	Primer outside *GAL4* downstream to confirm *kanMX* integration
oHLCH490	gcgttagaggtgctacagag	Amplify full *GAL4* F, no overhangs for transformation
oHLCH491	caccagcaaatatgagccatg	Amplify full *GAL4* R, no overhangs for transformation
oHLCH176	gacatggaggcccagaatac	Amplify *natMX* F for marker swap
oHLCH177	tcgacagcagtatagcgacc	Amplify *natMX* R for marker swap
oHLCH496	gatttttcccggggtgctcttcgcAAgagtgaaaaatttccaagagtc	Site directed mutagenesis of the Gal4p binding sites F
oHLCH497	agagcaccccgggaaaaatcagTTgagagtaaaccaccgtggtg	Site directed mutagenesis of the Gal4p binding sites R
oHLCH348	gcacgtcaagactgtcaagg	internal primer to confirm genomic safe haven integration R
oHLCH349	gattcgatactaacgccgcc	internal primer to confirm genomic safe haven integration F
oHLCH350	ttcctatatcgcctcctgagc	genomic safe haven upstream primer
oHLCH351	tctggtgaatcccatagcagc	genomic safe haven downstreamstream primer

*bold letters indicate the part that binds in primers with overhangs
** underline indicates mutated nucleotides

Appendix Table S2. Genetically modified strains made and used for this study.

Strain	Species	Description	Genotype
yHLCH149	*Sat. dispora*	*GAL4* deletion with *kanMX*	*gal4Δ::kanMX-1*
yHLCH151	*Sat. dispora*	*GAL4* deletion with *kanMX*	*gal4Δ::kanMX-19*
yHLCH155	*Sat. dispora*	*GAL4* deletion with *natMX*	*gal4Δ::natMX-2*
yHLCH156	*Sat. dispora*	*GAL4* deletion with *natMX*	*gal4Δ::natMX-4*
yHLCH169	*Sat. dispora*	GFP driven from *TDH* promoter in wild-type background	P_{TDH}*-GFP-kanMX*
yHLCH170	*Sat. dispora*	GFP driven from mutated *TDH* promoter in wild-type background	P_{tdh}*-UASG-GFP-kanMX*
yHLCH171	*Sat. dispora*	GFP driven from *TDH* promoter in *gal4Δ* background	*gal4Δ::natMX-2* P_{TDH}*-GFP-kanMX*
yHLCH172	*Sat. dispora*	GFP driven from mutated *TDH* promoter in gal4Δ background	*gal4Δ::natMX-2* P_{tdh}*-UASG-GFP-kanMX*
