# Supplementary figures and images for: Convergent evolution of aerobic fermentation through divergent mechanisms acting on key shared glycolytic genes
Source: EMBO J. 2026 Apr 10;45(10):3540–62. doi: 10.1038/s44318-026-00778-0 (PMC13187154; doi:10.1038/s44318-026-00778-0)

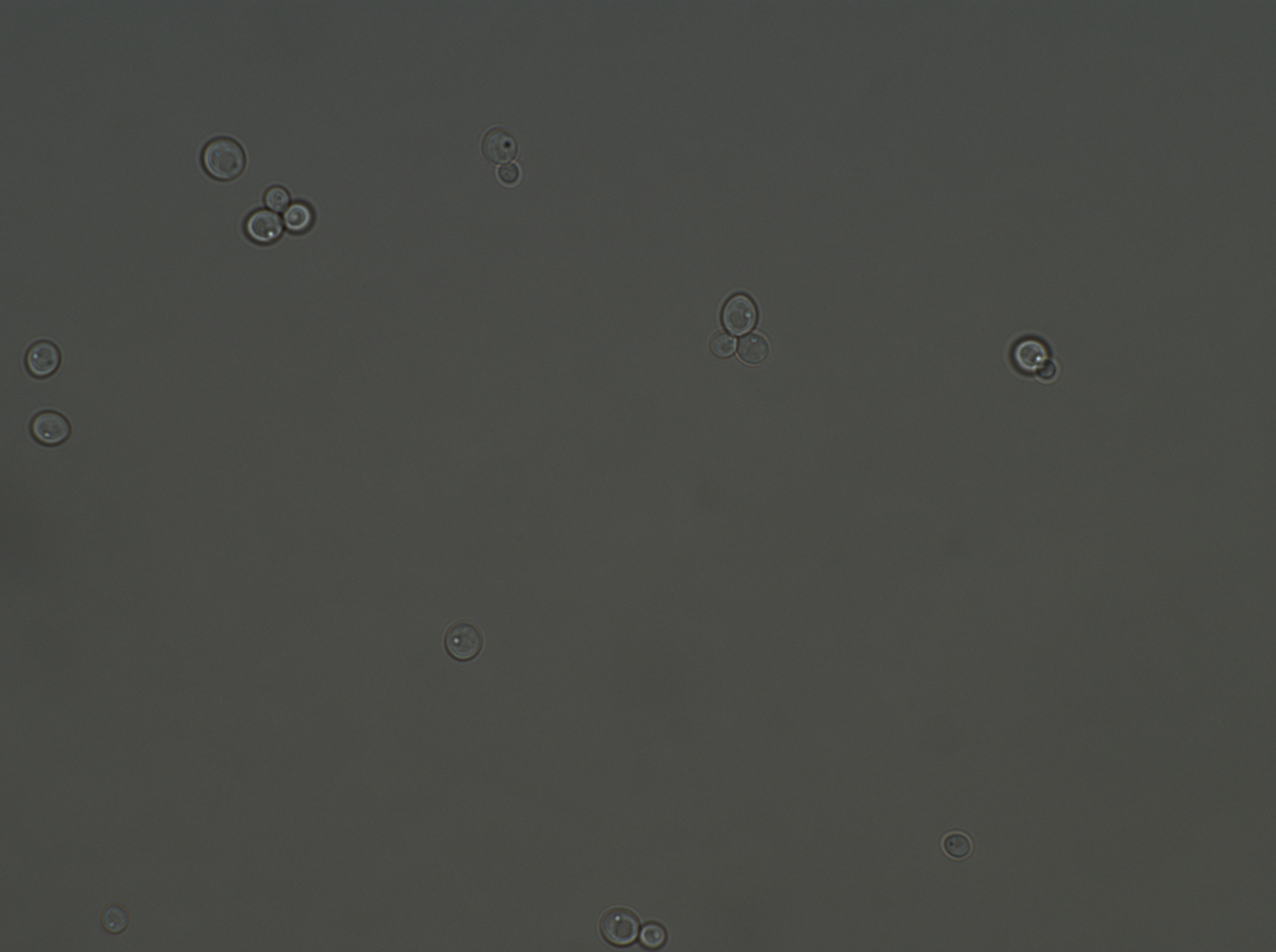

Supplement: Supplementary file 10 — Source data Fig. 5 [file 44318_2026_778_MOESM10_ESM.zip › Fig5_SD/Fig5C_Microscopy/gal4_PTDH-UASG_GFP_DIC_R_p00_0_A01f42d3.TIF]

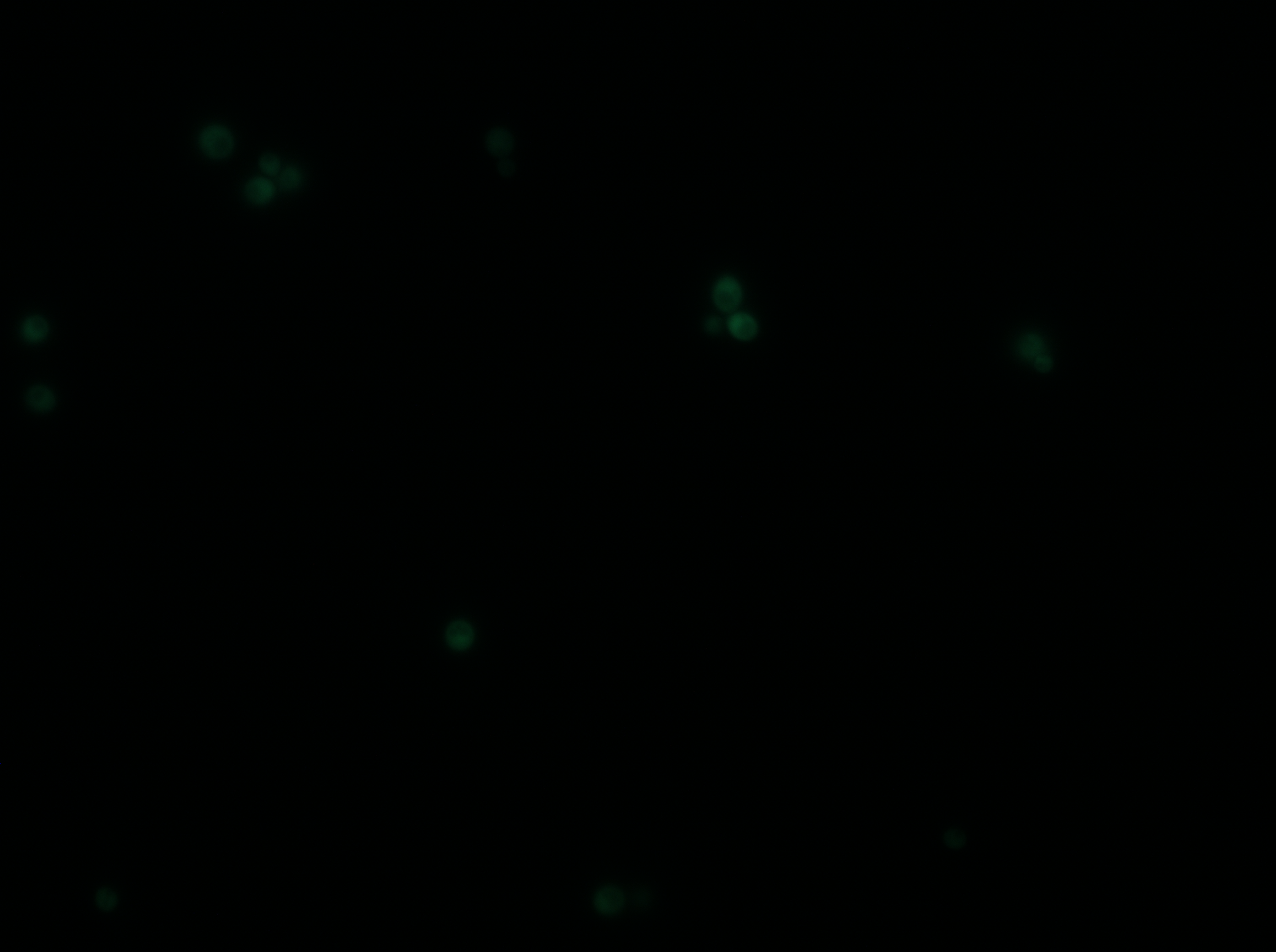

Supplement: Supplementary file 10 — Source data Fig. 5 [file 44318_2026_778_MOESM10_ESM.zip › Fig5_SD/Fig5C_Microscopy/gal4_PTDH-UASG_GFP_R_p00_0_A01f42d0.TIF]

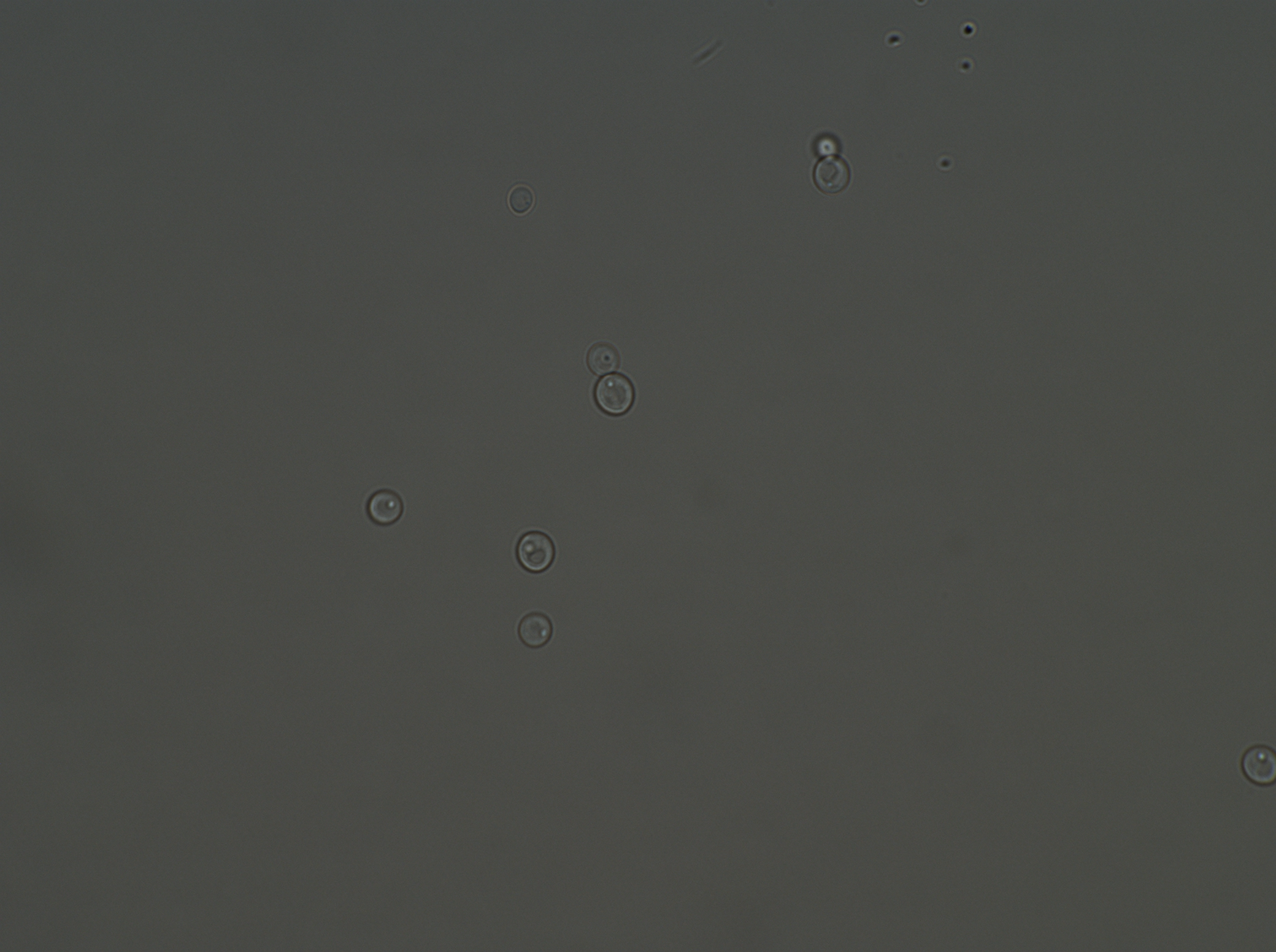

Supplement: Supplementary file 10 — Source data Fig. 5 [file 44318_2026_778_MOESM10_ESM.zip › Fig5_SD/Fig5C_Microscopy/gal4_PTDH_GFP_DIC_R_p00_0_A01f38d3.TIF]

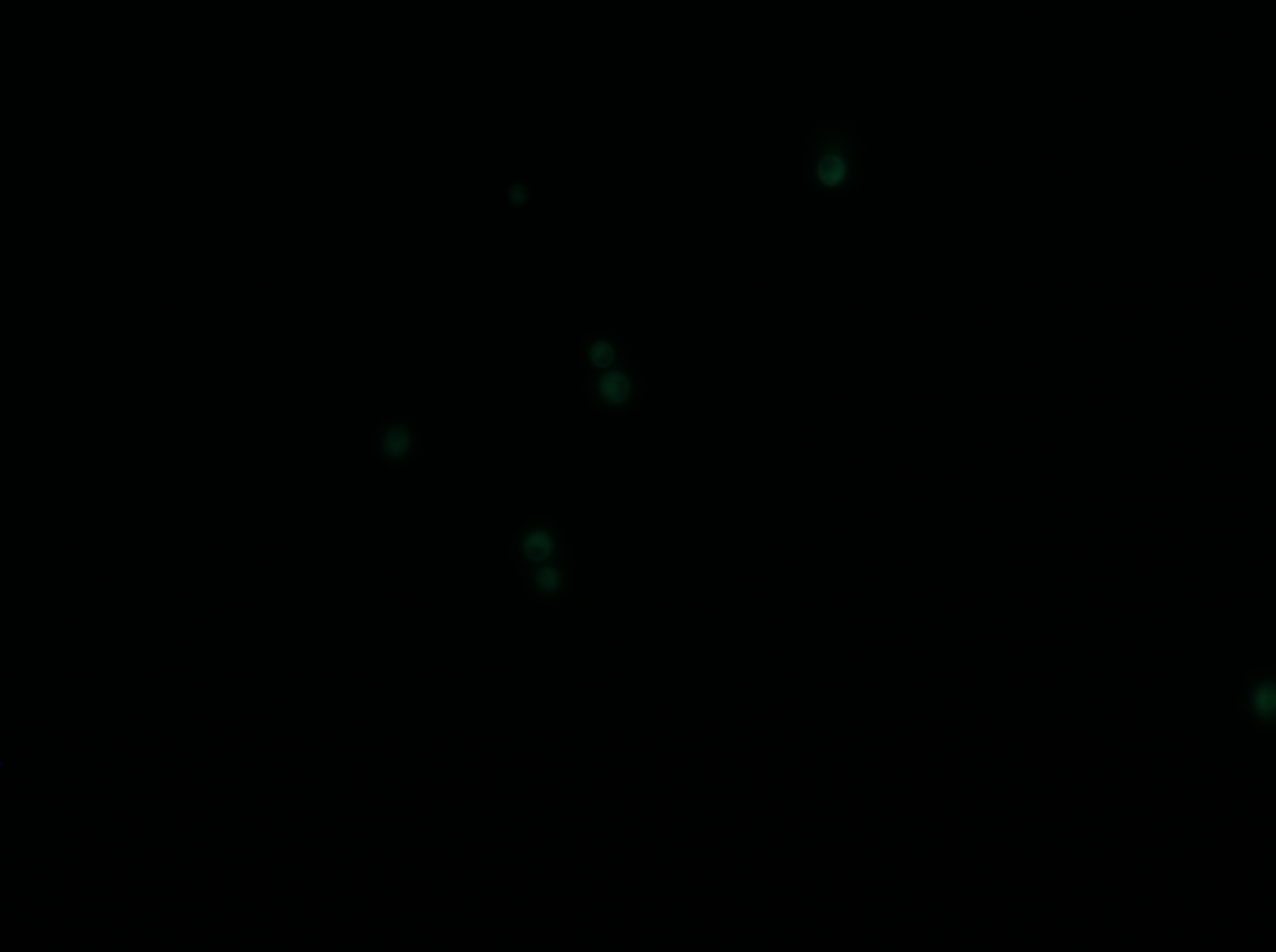

Supplement: Supplementary file 10 — Source data Fig. 5 [file 44318_2026_778_MOESM10_ESM.zip › Fig5_SD/Fig5C_Microscopy/gal4_PTDH_GFP_R_p00_0_A01f38d0.TIF]

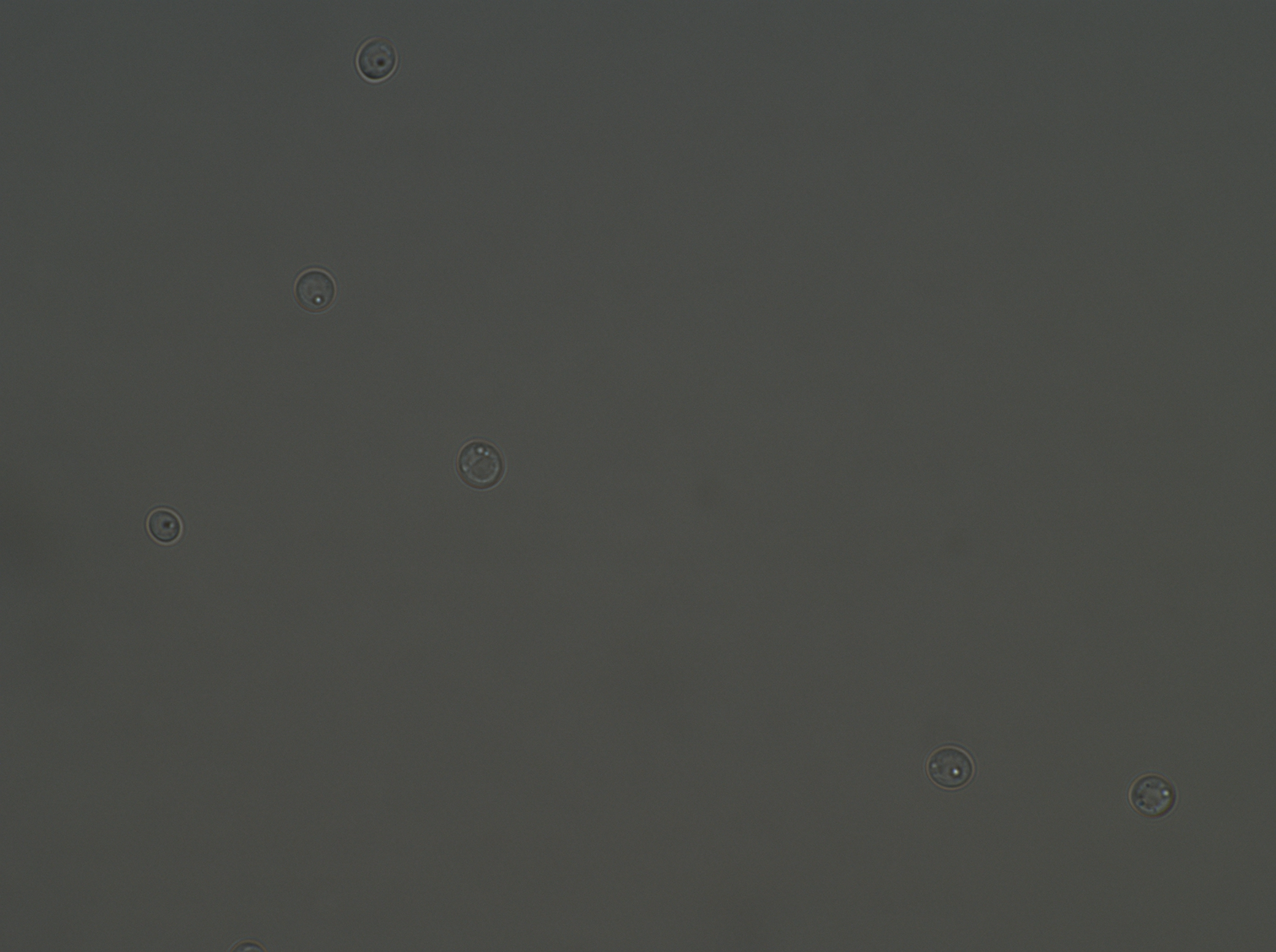

Supplement: Supplementary file 10 — Source data Fig. 5 [file 44318_2026_778_MOESM10_ESM.zip › Fig5_SD/Fig5C_Microscopy/WT_PTDH-UASG_GFP_DIC_R_p00_0_A01f17d3.TIF]

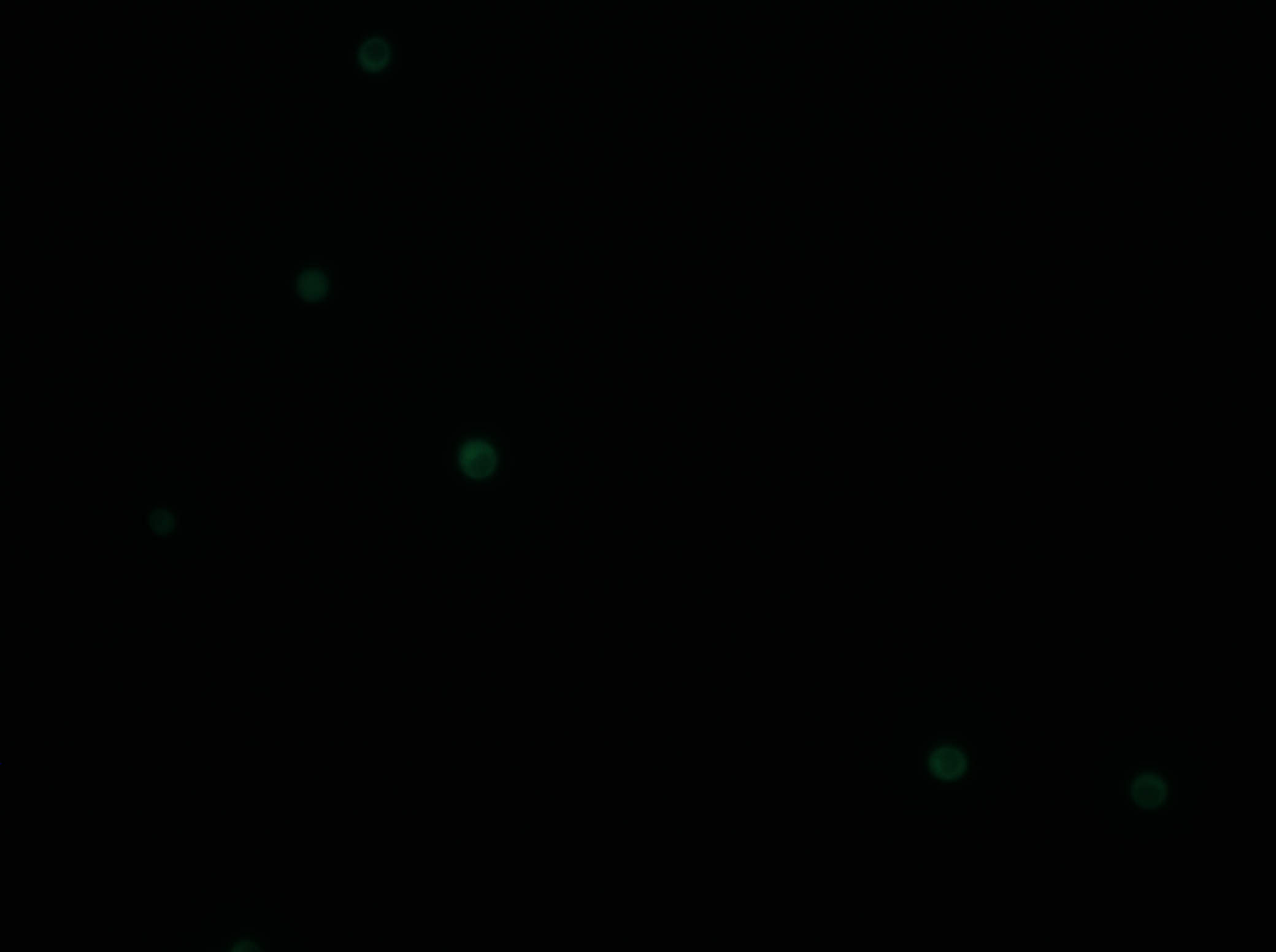

Supplement: Supplementary file 10 — Source data Fig. 5 [file 44318_2026_778_MOESM10_ESM.zip › Fig5_SD/Fig5C_Microscopy/WT_PTDH-UASG_GFP_R_p00_0_A01f17d0.TIF]

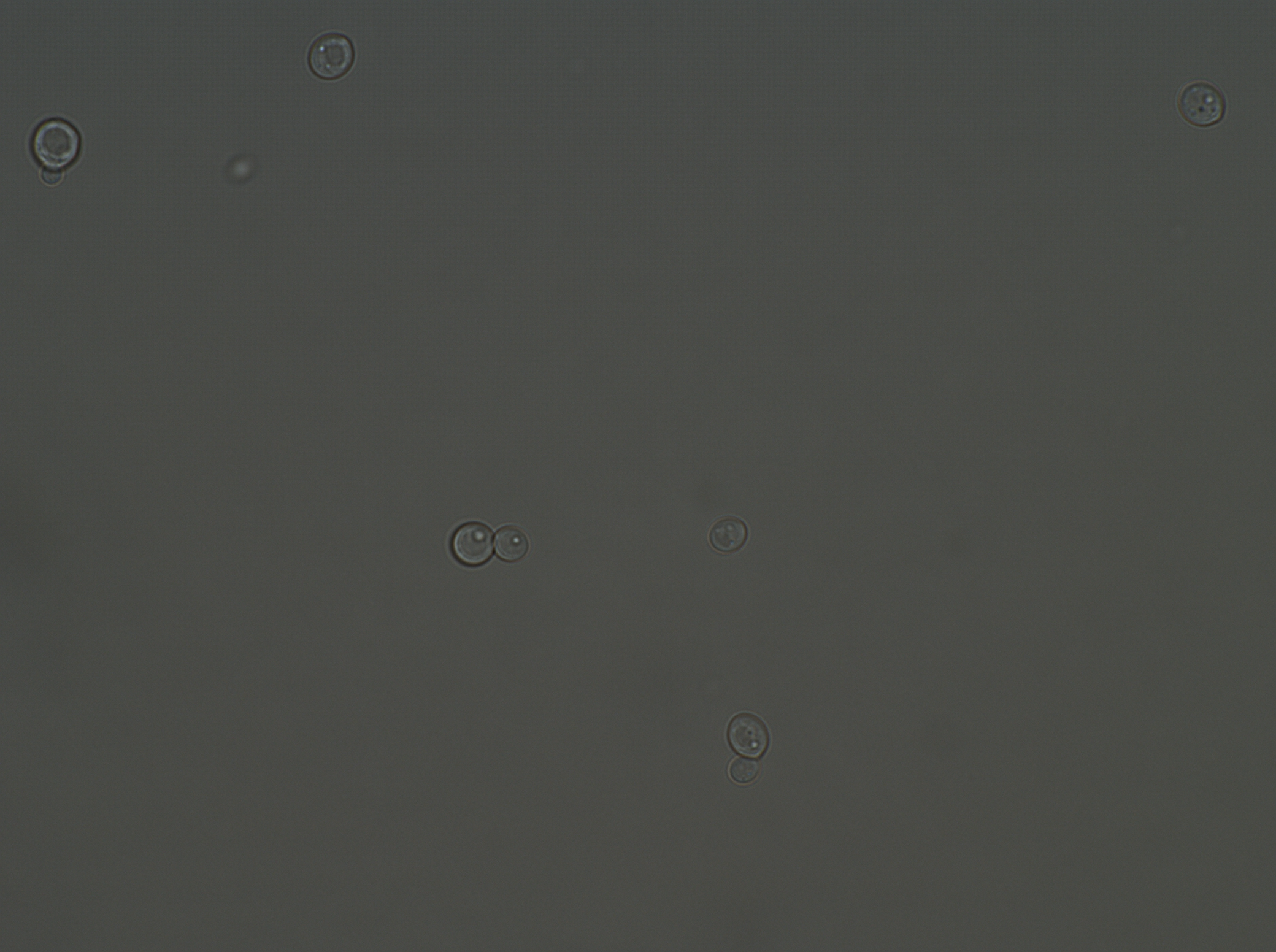

Supplement: Supplementary file 10 — Source data Fig. 5 [file 44318_2026_778_MOESM10_ESM.zip › Fig5_SD/Fig5C_Microscopy/WT_PTDH_GFP_DIC_R_p00_0_A01f03d3.TIF]

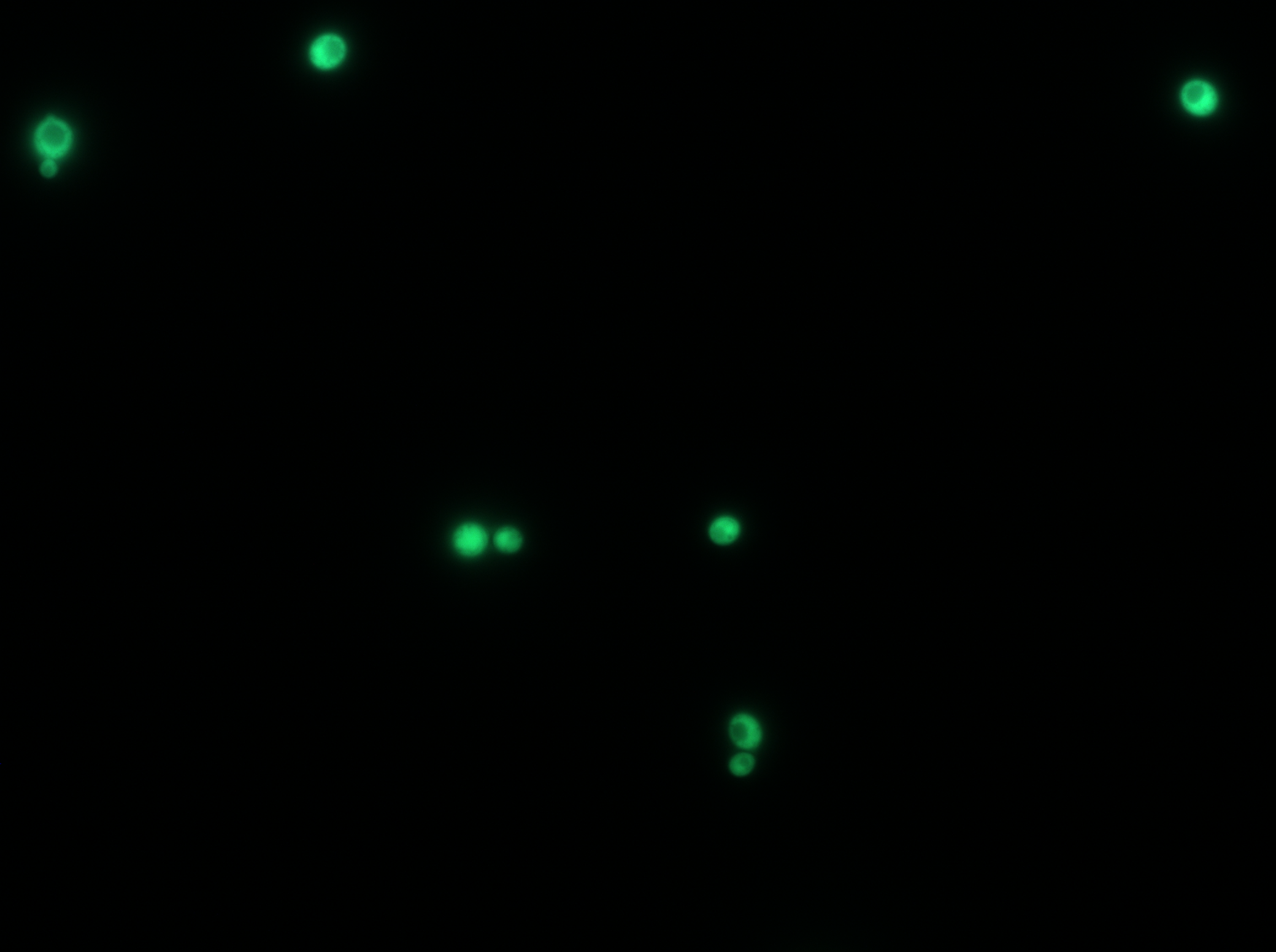

Supplement: Supplementary file 10 — Source data Fig. 5 [file 44318_2026_778_MOESM10_ESM.zip › Fig5_SD/Fig5C_Microscopy/WT_PTDH_GFP_R_p00_0_A01f03d0.TIF]
