# Supplementary material for: Convergent evolution of aerobic fermentation through divergent mechanisms acting on key shared glycolytic genes
Source: EMBO J. 2026 Apr 10;45(10):3540–62. doi: 10.1038/s44318-026-00778-0 (PMC13187154; doi:10.1038/s44318-026-00778-0)
Supplement: Supplementary file 12 — Expanded View Figures [file 44318_2026_778_MOESM12_ESM.pdf]

Expanded View Figures

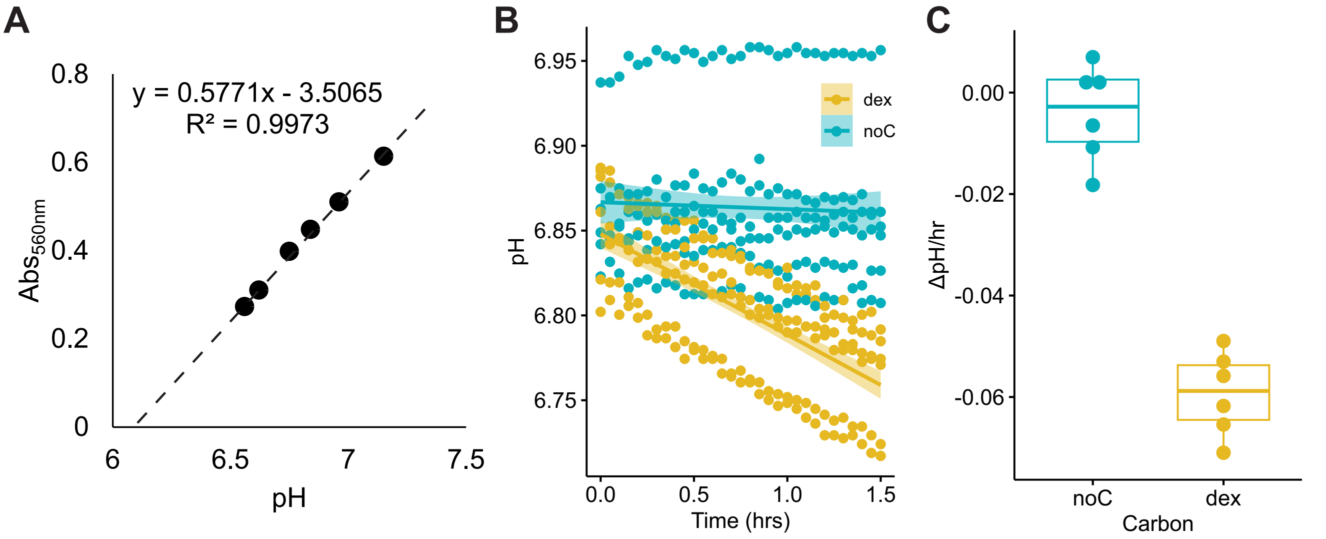

Figure EV1. Optimization of the extracellular acidification rate (ECAR) assay.

(**A**) A standard curve of the absorbance at 560 nm generated from media between pH 6.5 and 7.5. (**B**) Optimization assay using *S. cerevisiae* showing that the control with no carbon source (noC) did not acidify the media, whereas the cells with 2% glucose added (dex) consistently acidified the media across six biological replicates. (**C**) The slopes calculated from the six biological replicates showing the dextrose-dependent pH change. The center lines of boxplots represent the median, the bounds of the boxes represent the interquartile range, the whiskers represent the spread of the data, and each dot represents one of six biological replicates.

 © The Author(s)

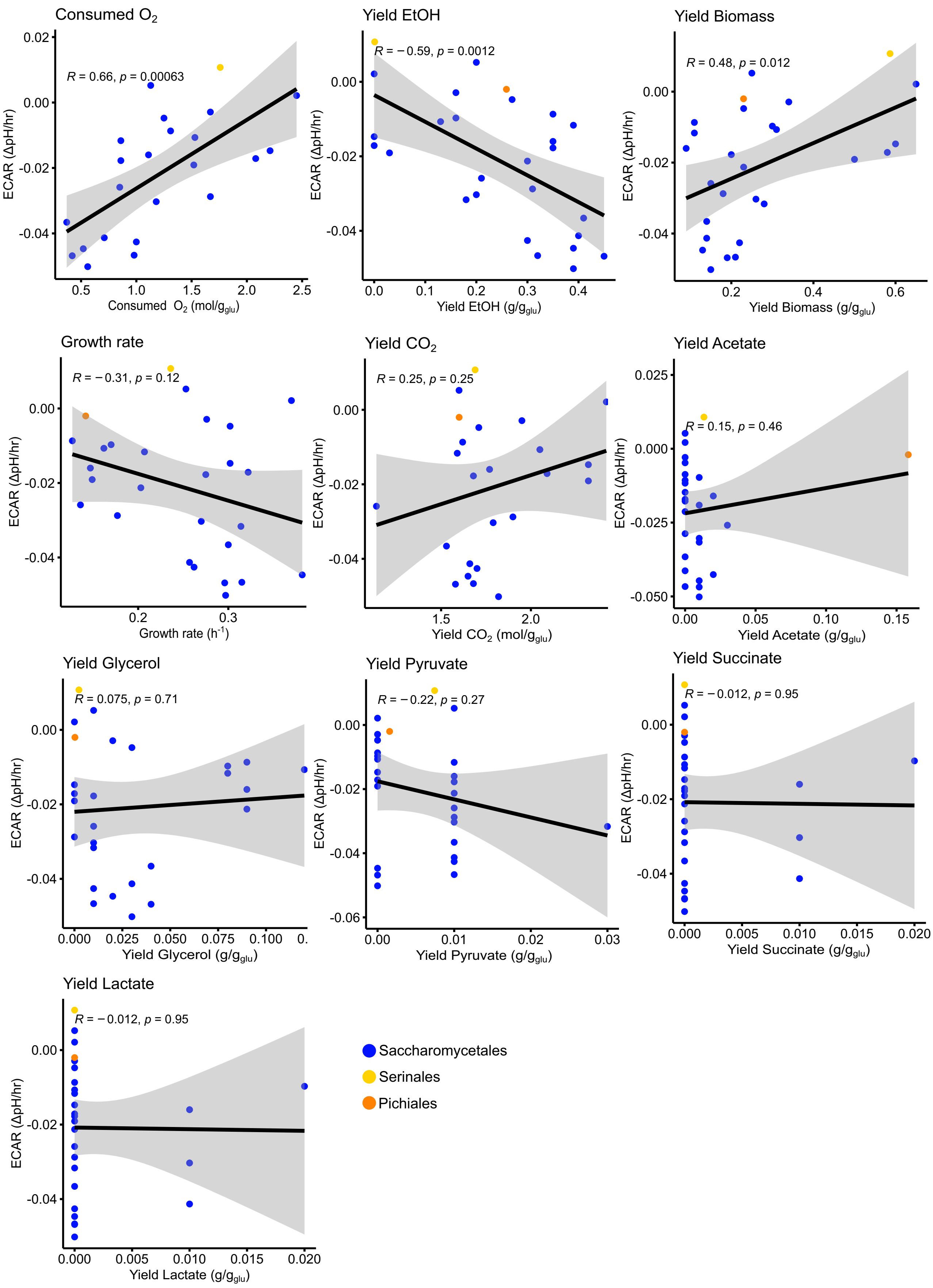
Consumed O2
R = 0.66, p = 0.00063
ECAR (ΔpH/hr)
0.02
0.00
-0.02
-0.04
0.5
1.0
1.5
2.0
2.5
Consumed O2 (mol/gglu)
Yield EtOH
R = −0.59, p = 0.0012
ECAR (ΔpH/hr)
0.00
-0.02
-0.04
0.0
0.1
0.2
0.3
0.4
Yield EtOH (g/gglu)
Yield Biomass
R = 0.48, p = 0.012
ECAR (ΔpH/hr)
0.00
-0.02
-0.04
0.2
0.4
0.6
Yield Biomass (g/gglu)
Growth rate
R = −0.31, p = 0.12
ECAR (ΔpH/hr)
0.00
-0.02
-0.04
0.2
0.3
Growth rate (h-1)
Yield CO2
R = 0.25, p = 0.25
ECAR (ΔpH/hr)
0.00
-0.02
-0.04
1.5
2.0
Yield CO2 (mol/gglu)
Yield Acetate
R = 0.15, p = 0.46
ECAR (ΔpH/hr)
0.025
0.000
-0.025
-0.050
0.00
0.05
0.10
0.15
Yield Acetate (g/gglu)
Yield Glycerol
R = 0.075, p = 0.71
ECAR (ΔpH/hr)
0.00
-0.02
-0.04
0.000
0.025
0.050
0.075
0.100
0.
Yield Glycerol (g/gglu)
Yield Pyruvate
R = −0.22, p = 0.27
ECAR (ΔpH/hr)
0.00
-0.02
-0.04
-0.06
0.00
0.01
0.02
0.03
Yield Pyruvate (g/gglu)
Yield Succinate
R = −0.012, p = 0.95
ECAR (ΔpH/hr)
0.00
-0.02
-0.04
0.000
0.005
0.010
0.015
0.020
Yield Succinate (g/gglu)
Yield Lactate
R = −0.012, p = 0.95
ECAR (ΔpH/hr)
0.00
-0.02
-0.04
0.000
0.005
0.010
0.015
0.020
Yield Lactate (g/gglu)
Saccharomycetales
Serinales
Pichiales

◀ **Figure EV2. Comparison of ECAR and parameters associated with the Crabtree/Warburg effect.**

The measured ECAR values were correlated with the parameters associated with ECAR collected by (Hagman et al, 2013). The R and *P* values are based on Pearson's correlations. Each dot represents a single species and is colored according to the taxonomic order of that species.

© The Author(s)

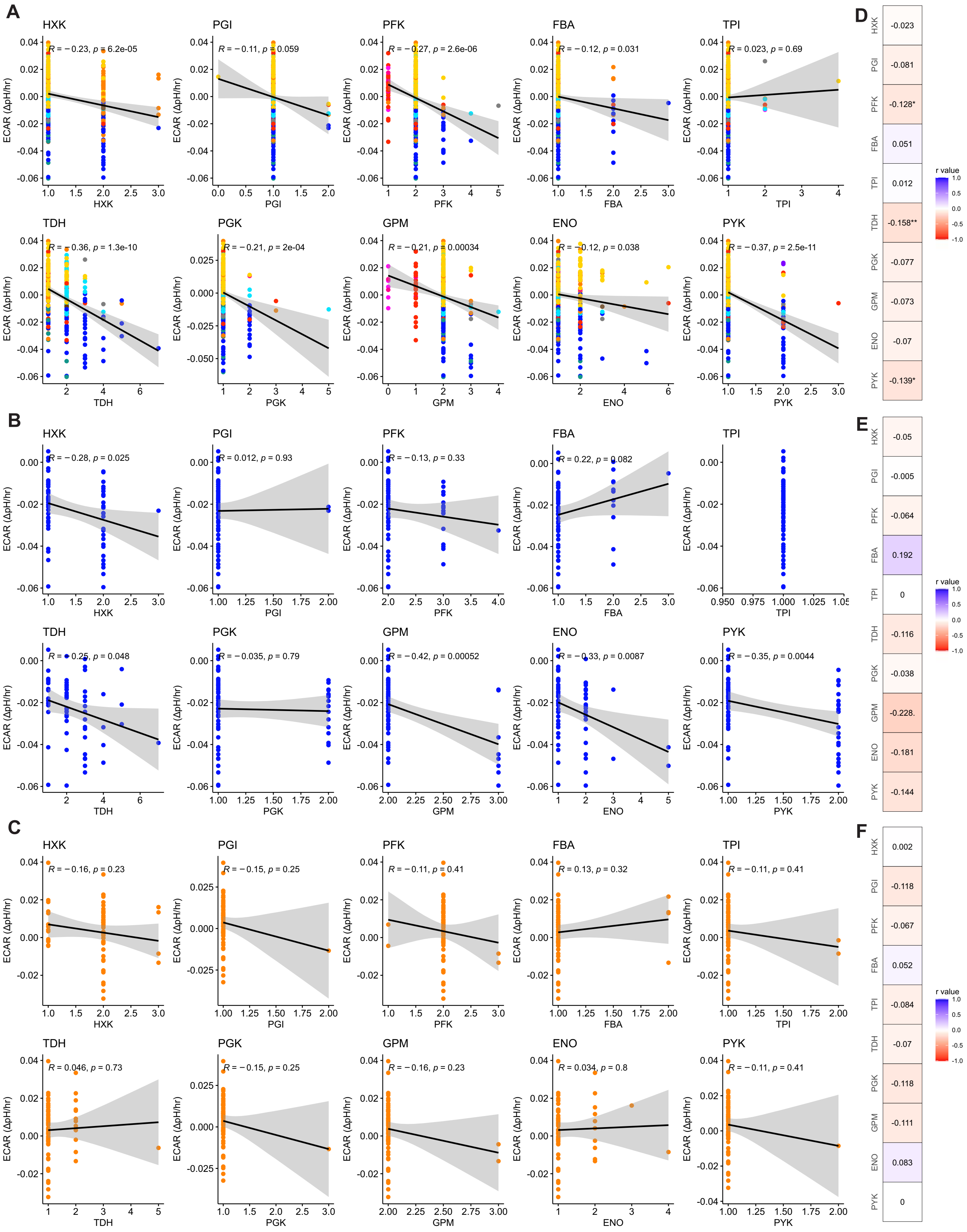
A
HXK
R = −0.23, p = 6.2e-05
PGI
R = −0.11, p = 0.059
PFK
R = −0.27, p = 2.6e-06
FBA
R = −0.12, p = 0.031
TPI
R = 0.023, p = 0.69
TDH
R = −0.36, p = 1.3e-10
PGK
R = −0.21, p = 2e-04
GPM
R = −0.21, p = 0.00034
ENO
R = −0.12, p = 0.038
PYK
R = −0.37, p = 2.5e-11
ECAR (ΔpH/hr)
B
HXK
R = −0.28, p = 0.025
PGI
R = 0.012, p = 0.93
PFK
R = −0.13, p = 0.33
FBA
R = 0.22, p = 0.082
TPI
TDH
R = 0.25, p = 0.048
PGK
R = −0.035, p = 0.79
GPM
R = −0.42, p = 0.00052
ENO
R = −0.33, p = 0.0087
PYK
R = −0.35, p = 0.0044
C
HXK
R = −0.16, p = 0.23
PGI
R = −0.15, p = 0.25
PFK
R = −0.11, p = 0.41
FBA
R = 0.13, p = 0.32
TPI
R = −0.11, p = 0.41
TDH
R = 0.046, p = 0.73
PGK
R = −0.15, p = 0.25
GPM
R = −0.16, p = 0.23
ENO
R = 0.034, p = 0.8
PYK
R = −0.11, p = 0.41
D
HXK -0.023
PGI -0.081
PFK -0.128*
FBA 0.051
TPI 0.012
TDH -0.158**
PGK -0.077
GPM -0.073
ENO -0.07
PYK -0.139*
r value
E
HXK -0.05
PGI -0.005
PFK -0.064
FBA 0.192
TPI 0
TDH -0.116
PGK -0.038
GPM -0.228.
ENO -0.181
PYK -0.144
F
HXK 0.002
PGI -0.118
PFK -0.067
FBA 0.052
TPI -0.084
TDH -0.07
PGK -0.118
GPM -0.111
ENO 0.083
PYK 0

Figure EV3. The gene family size of glycolytic genes correlates with ECAR across all yeasts.

(**A–C**) The correlation between ECAR and the number of homologs of each glycolytic gene showing the Pearson's correlation and *P* value for (**A**) all phenotyped yeasts, (**B**) the yeasts in the order Saccharomycetales, and (**C**) the yeasts in the order Pichiales. (**D–F**) The phylogenetically corrected correlations between ECAR and the number of homologs of each glycolytic gene for (**D**) all phenotyped yeasts, (**E**) the yeasts in the order Saccharomycetales, and (**F**) the yeasts in the order Pichiales. The *R* value from the phylogenetic generalized least squares is in the box and the *P* value is indicated as $*P < 0.05$ and $**P < 0.01$. Data points are color-coded according to taxonomic order as in Fig. 1.

© The Author(s)

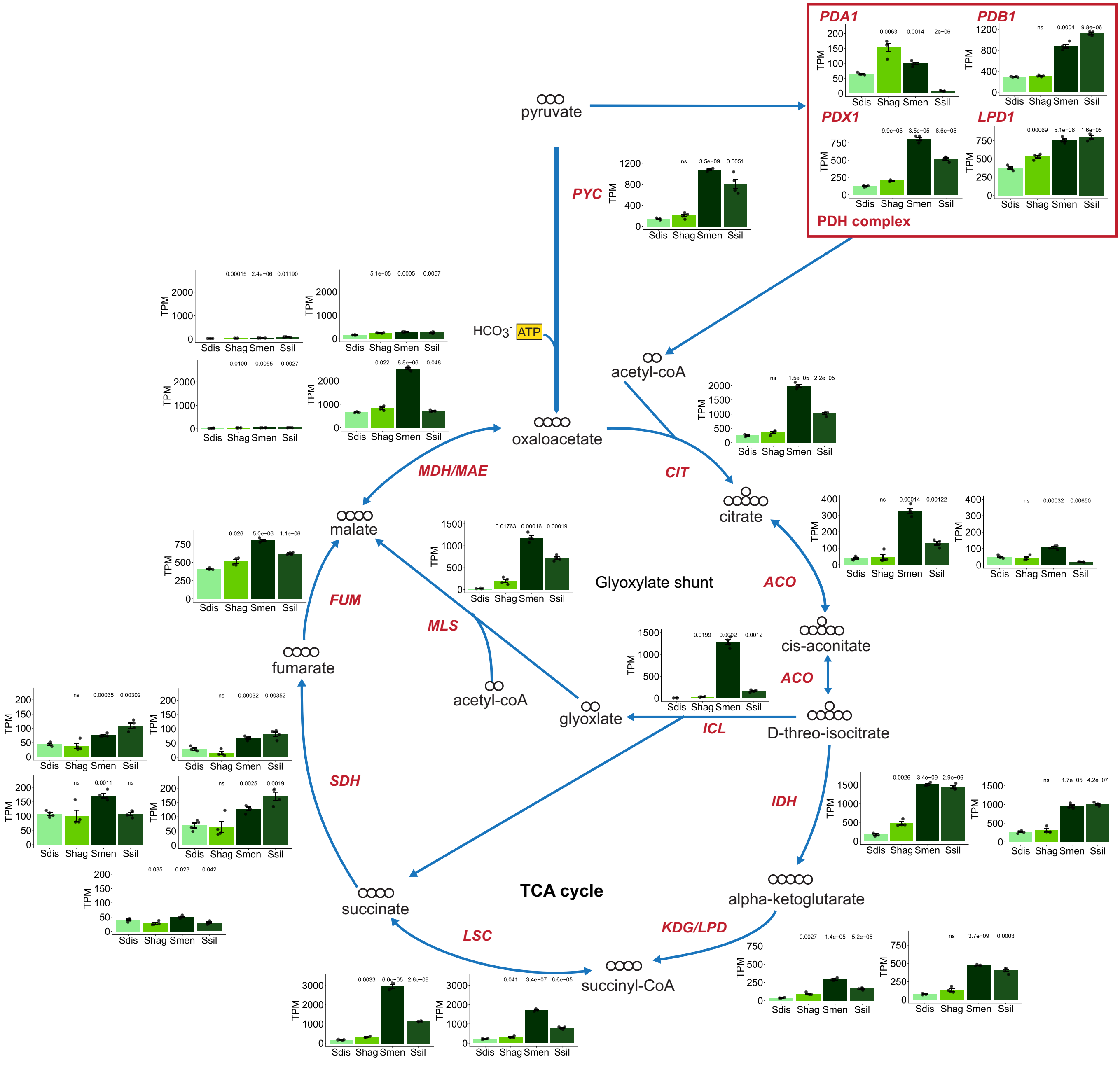

Figure EV4. Divergent expression patterns of TCA cycle genes between rapid ECAR and low ECAR *Saturnispora* species.

The gene expression for orthologous genes in the TCA cycle and glyoxylate shunt across two rapid ECAR (*Sat. dispora* Sdis, *Sat. hagleri* Shag) and two low ECAR (*Sat. mendoncae* Smen, *Sat. silvae* Ssil) are shown as transcripts per million (TPM). The bar height represents the average of four biological replicates: each replicate is shown as a black dot, and the error bars represent the standard error of the mean. The significance is relative to the TPM in *Sat. dispora* (ns not significant). In the schematic of glycolysis, carbon backbones are white.

© The Author(s)

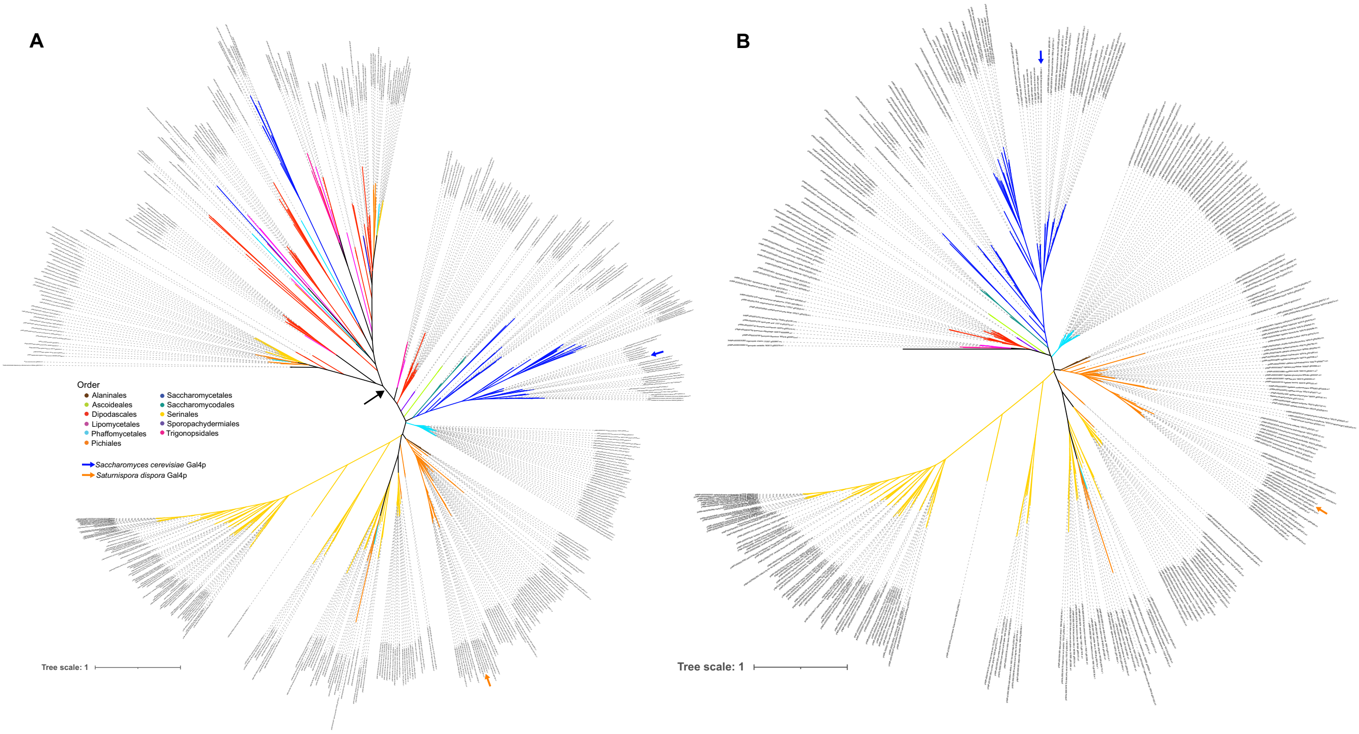

Figure EV5. Unrooted phylogeny of the Gal4p orthologs in the budding yeasts used in this study.

(**A**) A phylogeny including all translated amino acid sequences of genes in the orthogroup containing canonical Gal4p. A black arrow indicates the location of a branch, which was pruned in (**B**) due to the length of the branch and the topological incongruence with the species phylogeny in this part of the tree, suggesting that these are likely other related C6 zinc transcription factors. The Newick files for these trees are available as source data. The location of the Gal4p orthologs from *S. cerevisiae* and *Sat. dispora* are highlighted on both trees, and the branches are colored according to the taxonomic order. Source data are available online for this figure.

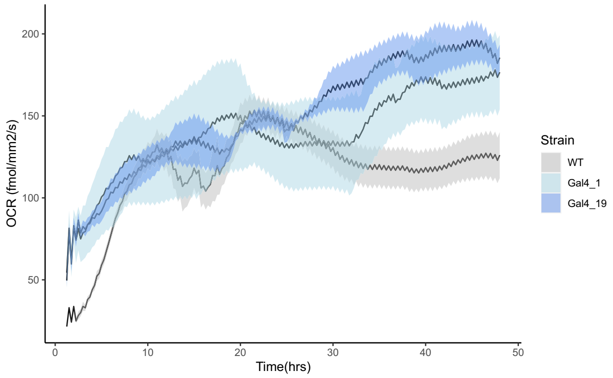

Figure EV6. The respiration profile of *Sat. dispora* and *gal4Δ* deletion mutants over 48 h of growth.

For each strain, the black line represents the average oxygen consumption rate (OCR), and the shaded area represents the standard error of the mean. Note that both mutants (Gal4_1, Gal4_19) have considerably higher OCR at several phases of the experiment, including the beginning and end.
